# Targeting the canonical ER stress IRE1α/XBP1 pathway counteracts pancreatic cancer-induced skeletal muscle wasting

**DOI:** 10.1101/2025.05.05.652304

**Published:** 2025-05-05

**Authors:** Aniket S. Joshi, Meiricris Tomaz da Silva, Anh Tuan Vuong, Bowen Xu, Ravi K. Singh, Ashok Kumar

## Abstract

Cancer-driven cachexia is a deleterious syndrome which involves progressive loss of skeletal muscle mass with or without fat loss, fatigue, and weakness that cannot be reversed by nutritional intake. Recent studies have shown deregulation of endoplasmic reticulum (ER)-induced unfolded protein response (UPR) pathways in skeletal muscles in various catabolic conditions, including cancer growth. However, the role of individual arms of the UPR in regulation of muscle mass remains poorly understood. Here, we demonstrate that the IRE1α/XBP1 arm of the UPR stimulates the activation of ubiquitin-proteasome system, autophagy, JAK-STAT3 signaling, and fatty acid oxidation in skeletal muscle of the KPC mouse model of pancreatic cancer cachexia. Furthermore, our results show that IRE1α/XBP1 pathway is a key contributor to cachexia as targeted ablation of XBP1 transcription factor in mouse skeletal muscle inhibits KPC tumor-induced muscle wasting. Transcriptionally active XBP1 protein binds to the promoter region of multiple genes whose products are involved in skeletal muscle wasting. Treatment of KPC tumor-bearing mice with 4µ8C, a small molecule IRE1α inhibitor, reverses cachexia-induced molecular changes and improves skeletal muscle mass and strength. Altogether, our study highlights that the IRE1α/XBP1 signaling axis mediates pancreatic cancer-induced muscle wasting and inhibition of this pathway could be a potential approach to mitigate muscle wasting in pancreatic cancer patients.

## Full Text Availability

The license terms selected by the author(s) for this preprint version do not permit archiving in PMC. The full text is available from the preprint server.

