# Supplementary material for: The canonical ER stress IRE1α/XBP1 pathway mediates skeletal muscle wasting during pancreatic cancer cachexia
Source: bioRxiv. 2025 Aug 4:2025.05.05.652304. Originally published 2025 May 5. Preprint. [Version 2] doi: 10.1101/2025.05.05.652304 (PMC12247886; doi:10.1101/2025.05.05.652304)
Supplement: Supplement 1 [file media-1.pdf]

Supplemental Data File

Targeting the canonical ER stress IRE1α/XBP1 pathway counteracts pancreatic cancer-induced skeletal muscle wasting

by

Aniket S. Joshi, Meiricris Tomaz da Silva, Anh Tuan Vuong, Bowen Xu, Ravi K. Singh, and Ashok Kumar

This file contains Supplemental Figures S1-S6 and Tables S1-S3

SUPPLEMENTAL FIGURES

FIGURE S1

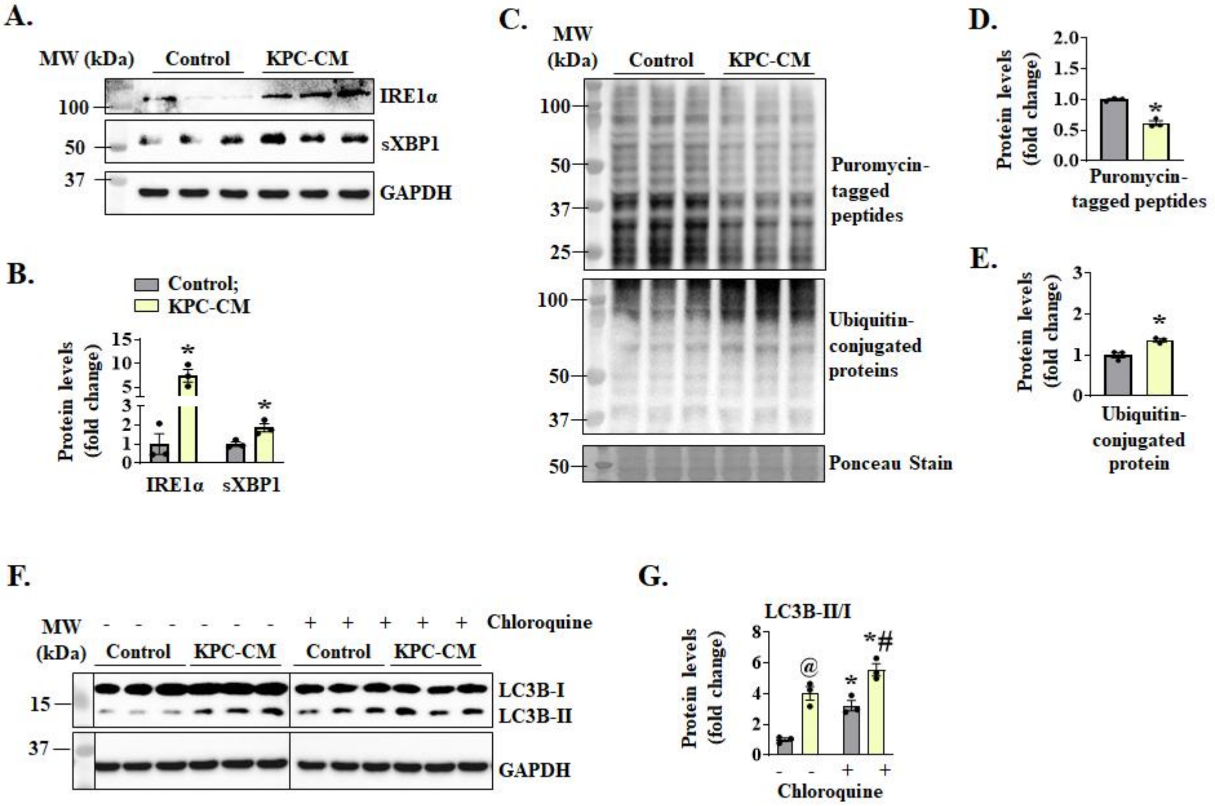

Figure S1. KPC tumor-derived factors activate IRE1α/XBP1 signaling and regulate protein turnover in cultured myotubes. Mouse primary myotubes were treated with conditioned media from KPC cells (KPC-CM) or with exhausted differentiation media (as control). After incubation for 24 h, the cells were collected for western blot analysis. **(A)** Immunoblots and **(B)** densitometry analysis of levels of IRE1α and sXBP1 protein in control and KPC-CM treated myotube cultures. **(C)** Immunoblots, and densitometry analysis of levels of **(D)** puromycin-tagged peptides and **(E)** Ubiquitin-conjugated proteins in control and KPC-CM treated cultures. n=3 biological replicates per group. All data are presented as mean ± SEM. *$p < 0.05$, values significantly different from corresponding control cultures; analyzed by unpaired Student *t* test. After 24 h treatment with DM or KPC-CM, 100µM chloroquine or vehicle alone was added to myotube culture media for 1 h, followed by collection of the cells for western blotting. **(F)** Immunoblot and **(G)** quantification of ratio of LC3B-II/I proteins in control and KPC-CM treated myotube cultures treated with vehicle alone or chloroquine. n=3 biological replicates per

group. All data are presented as mean ± SEM. *$p < 0.05$, values significantly different from corresponding cultures treated with vehicle alone; $^{@}p<0.05$, values significant different from control cultures treated with vehicle alone. $^{\#}p < 0.05$, values significantly different from control cultures treated with chloroquine, analyzed by two-way ANOVA followed by Tukey's multiple comparison test.

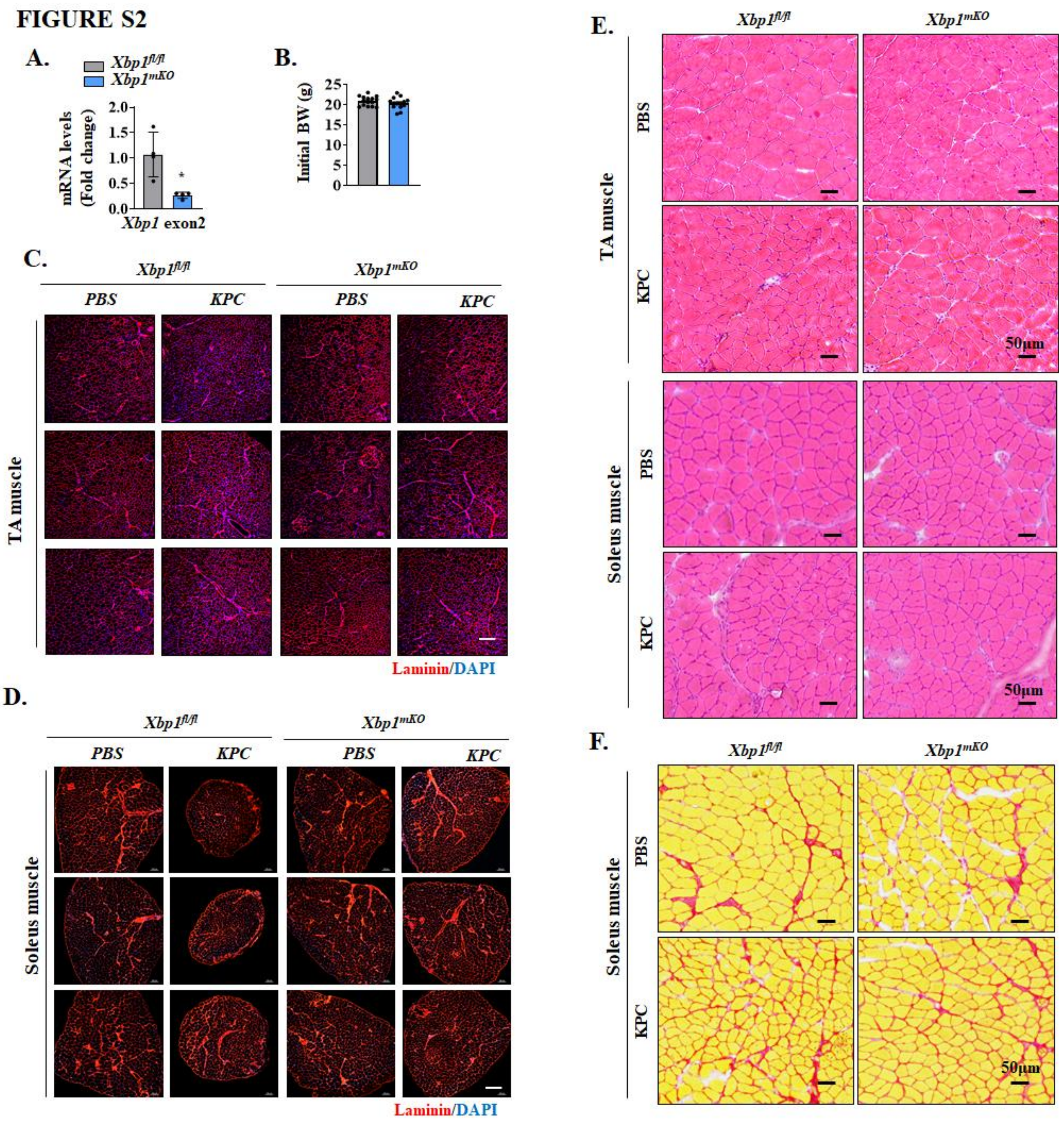

Figure S2. Targeted deletion of XBP1 inhibits muscle atrophy in KPC tumor-bearing mice. **(A)** Relative mRNA levels of XBP1 determined by qRT-PCR analysis using primer set specific for Xbp1 exon2, a sequence flanked by the loxP sites, in gastrocnemius (GA) muscle of $Xbp1^{fl/fl}$ and $Xbp1^{mKO}$ mice. n=4 mice per group. Data are presented as mean ± SEM. *$p < 0.05$, values significantly different from GA muscle of $Xbp1^{fl/fl}$ mice, analyzed by unpaired Student *t* test. **(B)** Quantification of initial body weight of $Xbp1^{fl/fl}$ and $Xbp1^{mKO}$ mice. n=14-15 mice per group. No

significant difference was observed using unpaired Student *t* test. Transverse sections of TA and soleus muscle isolated from PBS- or KPC cells-injected *Xbp1*$^{fl/fl}$ and *Xbp1*^{mKO} mice were used for anti-laminin and DAPI staining or H&E staining. Anti-laminin and DAPI stained sections of **(C)** TA and **(D)** soleus muscle from multiple mice. Scale bar, 200μm. **(E)** Representative photomicrographs of H&E-stained transverse sections of TA (upper panel) and soleus (lower panel) muscle of control and KPC tumor-bearing *Xbp1*$^{fl/fl}$ and *Xbp1*^{mKO} mice. Scale bar, 50μm. **(F)** Representative photomicrographs of Sirius red-stained soleus muscle sections of *Xbp1*$^{fl/fl}$ and *Xbp1*^{mKO} mice injected with PBS or KPC cells. Scale bar, 50μm.

FIGURE S3

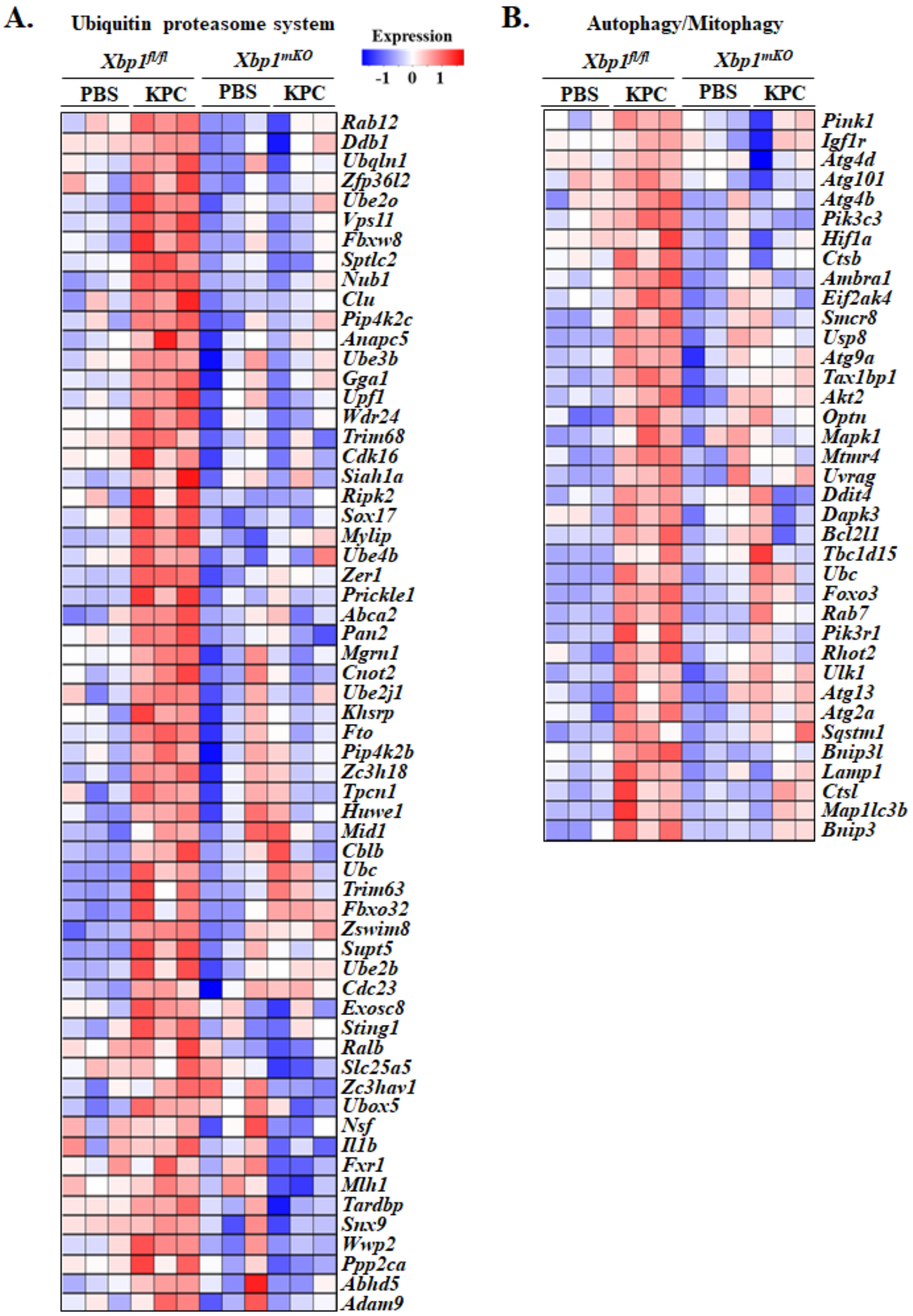

Figure S3. Targeted ablation of XBP1 inhibits the expression of multiple genes involved in proteolysis. Heatmap representation of RNA-Seq dataset analysis showing relative expression of genes involved in **(A)** Ubiquitin proteasome system (UPS), and **(B)** autophagy/mitophagy in gastrocnemius muscle of control and KPC tumor-bearing *Xbp1*$^{fl/fl}$ and *Xbp1*^{mKO} mice.

FIGURE S4

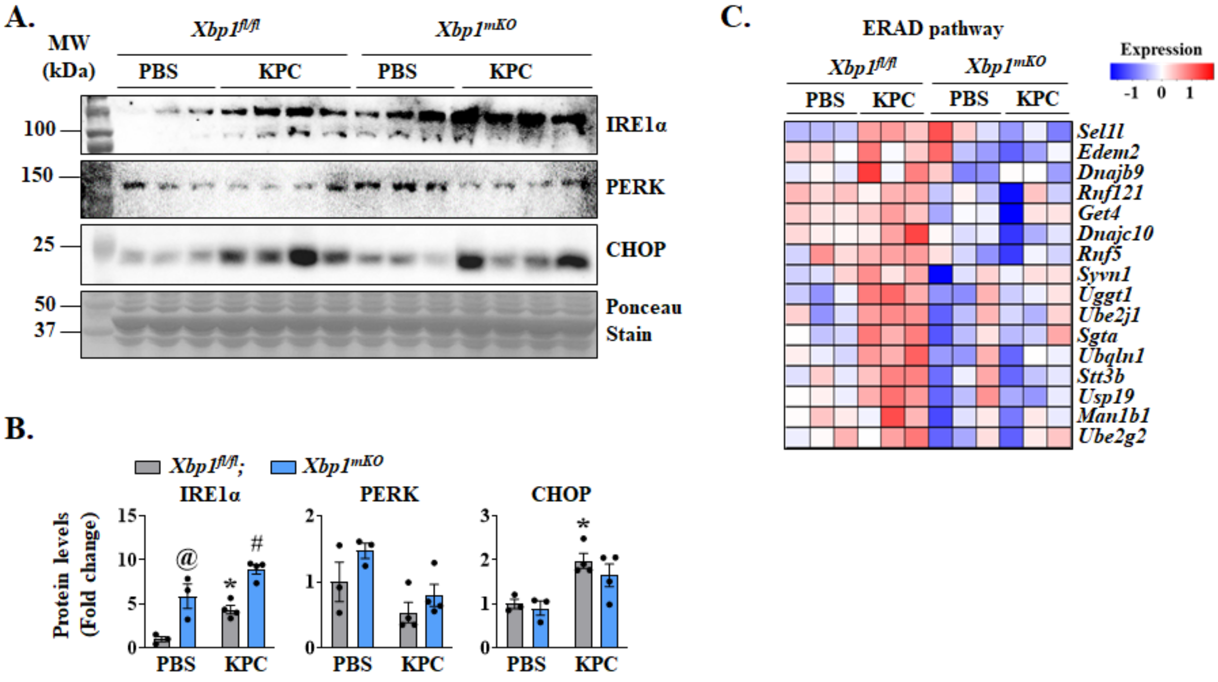

Figure S4. Effect of targeted ablation of XBP1 on levels of UPR markers. (A) Immunoblots and **(B)** densitometry analysis of protein levels of IRE1α, PERK, and CHOP in gastrocnemius (GA) muscle of *Xbp1*$^{fl/fl}$ and *Xbp1*^{mKO} mice injected with PBS or KPC cells. n=3-4 mice per group. Data are presented as mean ± SEM. $^{@}p < 0.05$, values significantly different from PBS-injected *Xbp1*$^{fl/fl}$ mice; $^{*}p < 0.05$, values significantly different from corresponding PBS-injected mice; $^{\#}p < 0.05$, values significantly different from KPC tumor-bearing *Xbp1*$^{fl/fl}$ mice, analyzed by two-way ANOVA followed by Tukey's multiple comparison test. **(C)** Heatmap representing relative gene expression of various molecules involved in ER associated degradation (ERAD) pathway in GA muscle of control and KPC tumor-bearing *Xbp1*$^{fl/fl}$ and *Xbp1*^{mKO} mice analyzed using bulk RNA-seq dataset.

FIGURE S5

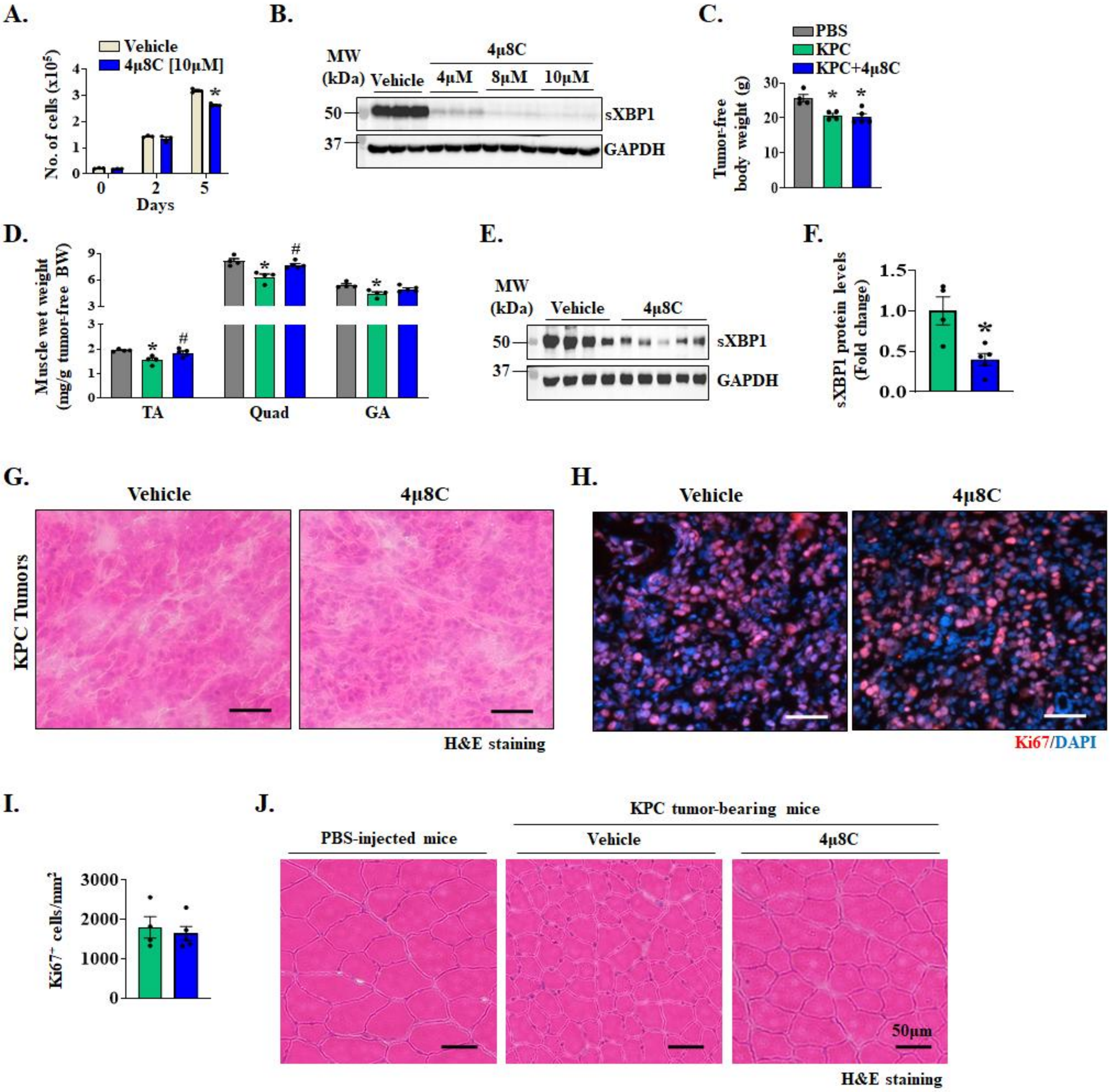

Figure S5. Effect of pharmacological inhibition of IRE1α/XBP1 axis on cancer cachexia. **(A)** Quantification of number of proliferating KPC cells on day 0, 2 and 5 after treatment with vehicle alone or 10 μM 4μ8C. n=3 biological replicates per group. Data are presented as mean ± SEM. *$p < 0.05$, values significantly different from vehicle-treated KPC cultures on day 5, analyzed by unpaired Student *t* test. **(B)** Immunoblot showing levels of sXBP1 protein in KPC cells treated with vehicle alone or indicated concentrations of 4μ8C for 24 h. **(C)** Quantification of tumor-free body weight (BW) of control and KPC tumor-bearing mice treated with vehicle

alone or 4μ8C after 18 days of KPC cells injection into the pancreas. **(D)** Quantification of wet weight of TA, Quad, and GA muscle normalized by tumor-free BW. n=4-5 mice per group. Data are presented as mean ± SEM. $^{*}p < 0.05$, values significantly different from PBS-injected mice; $^{\#}p < 0.05$, values significantly different from vehicle-treated KPC tumor-bearing mice, analyzed by one-way ANOVA, followed by Tukey's multiple comparison test. **(E)** Immunoblot and **(F)** densitometry analysis showing levels of sXBP1 protein in KPC tumors of mice treated with vehicle alone or 4μ8C. Data are presented as mean ± SEM. $^{*}p < 0.05$, values significantly different from vehicle-treated KPC tumor-bearing mice, analyzed by unpaired Student *t* test. Representative photomicrographs of KPC tumors after **(G)** H&E staining, or **(H)** anti-Ki67 and DAPI staining. Scale bar, 50μm. **(I)** Quantification of number of $Ki67^{+}$ cells per unit area (mm^2) in KPC tumors of mice treated with vehicle alone of 4μ8C. No significant difference was observed using unpaired Student *t* test. **(J)** Transverse sections of TA muscle of control and vehicle or 4μ8C-treated KPC tumor-bearing mice were generated and used for H&E staining. Representative photomicrographs of H&E-stained sections are presented here. Scale bar, 50μm.

FIGURE S6

Fig.1I

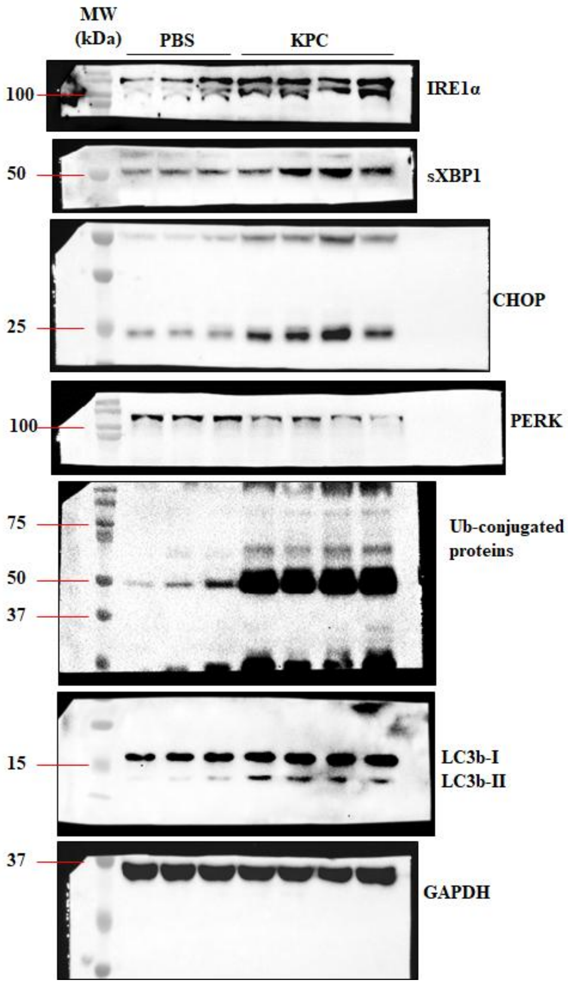

Fig.3D

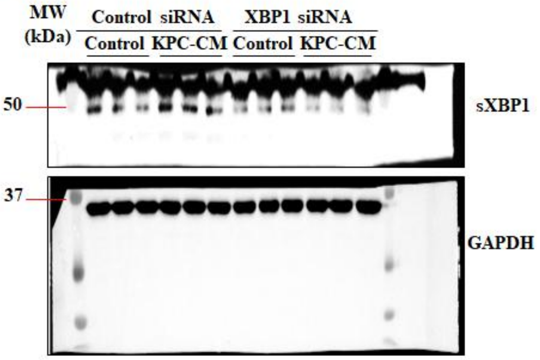

Fig.3J

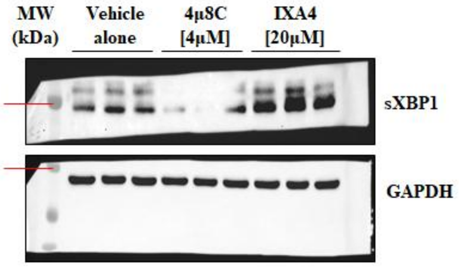

FIGURE S6 (continued)

Fig. 4C

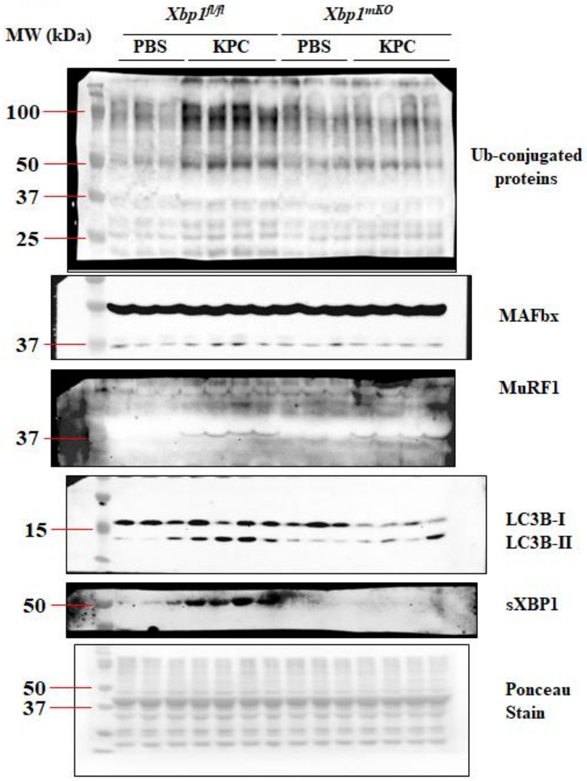

Fig. 4E

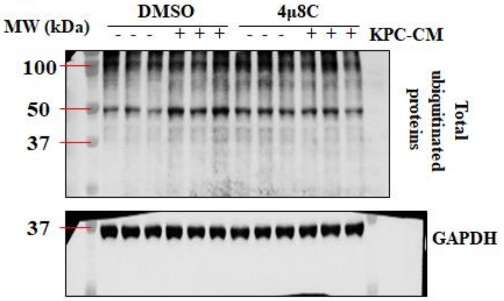

Fig. 5C

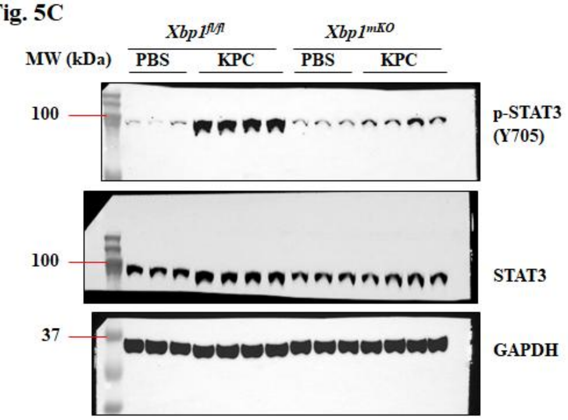

Fig. 5E

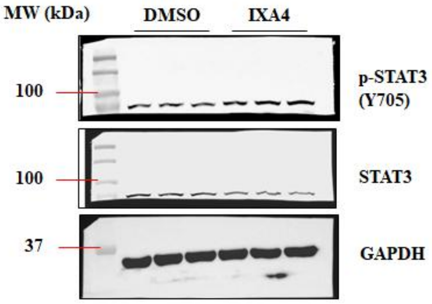

Fig. 5G

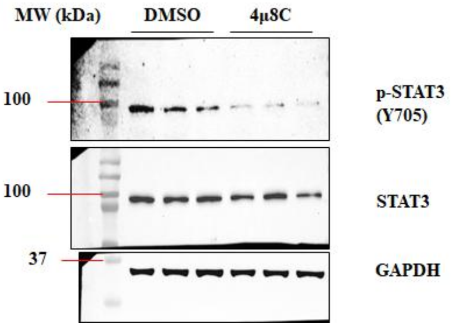

FIGURE S6 (continued)

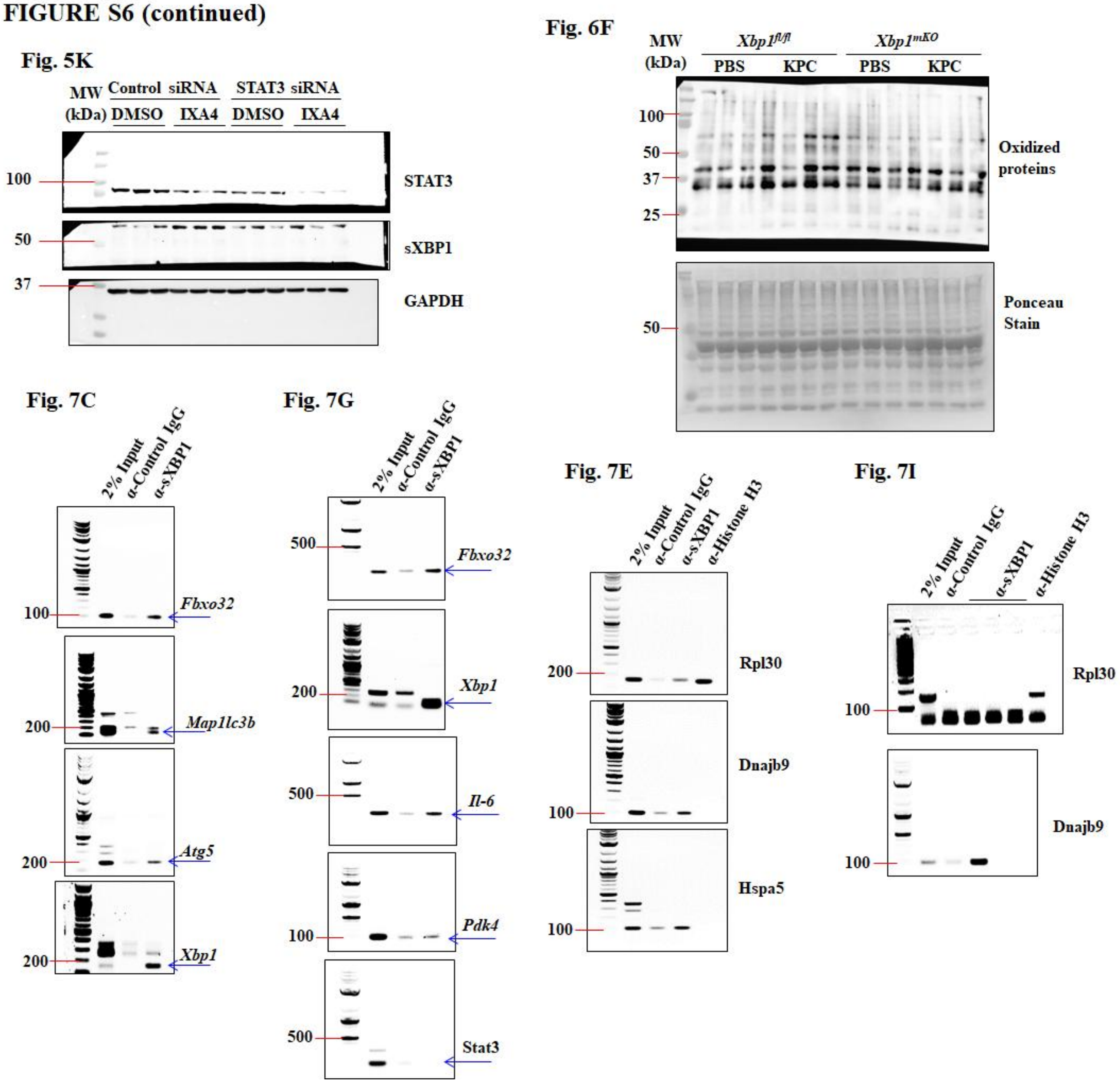

FIGURE S6 (continued)

Fig. 8I

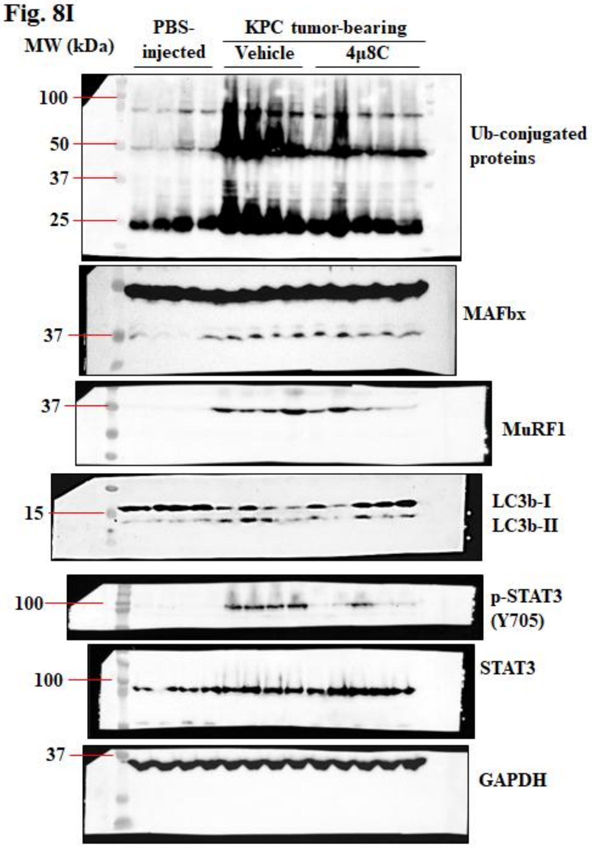

Fig. 8K

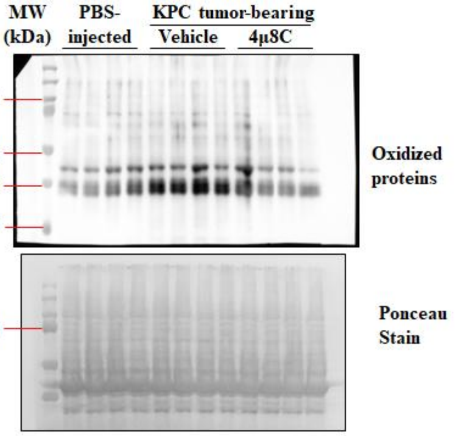

Fig. S1A

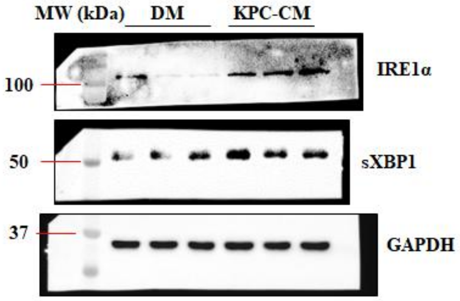

Fig. S1C

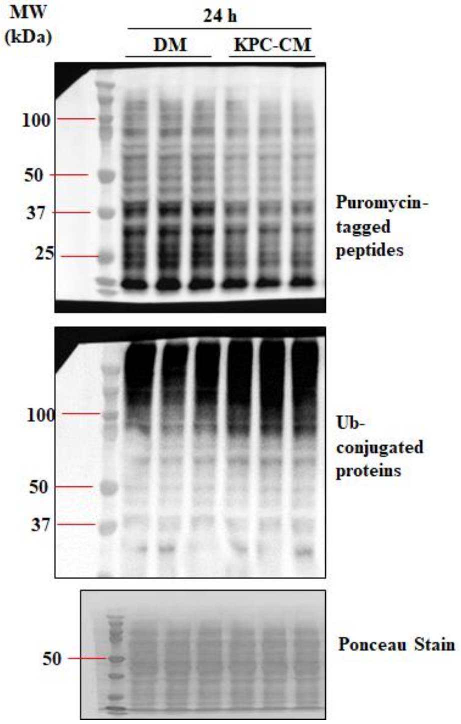

FIGURE S6 (continued)

Fig. S1F

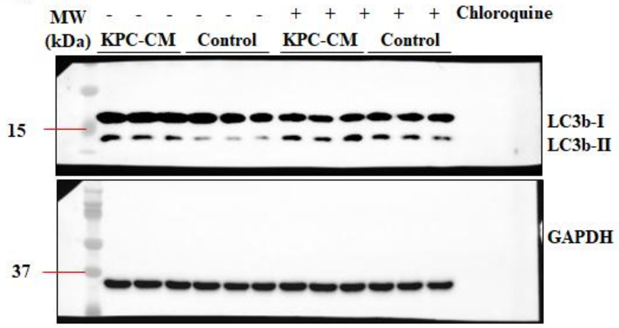

Fig. S4A

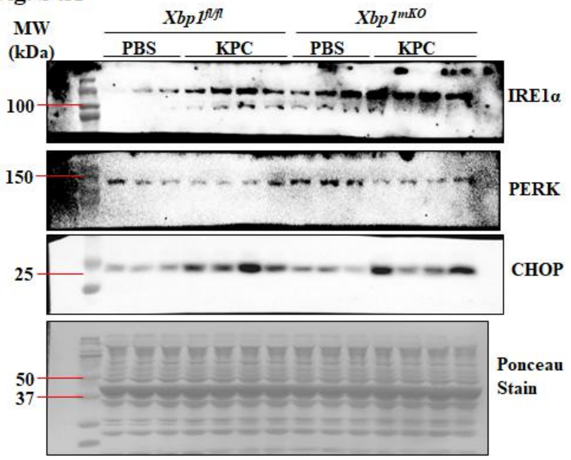

Fig. S5B

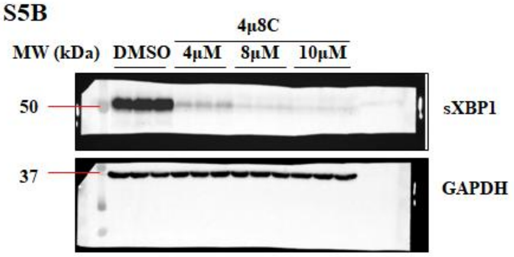

Fig. S5E

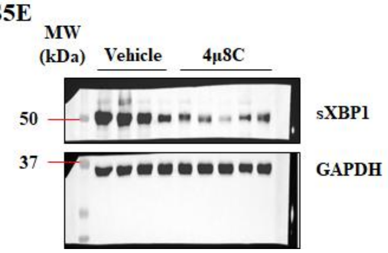

Figure S6. Uncropped western blot and agarose gel images.

Table S1. List of antibodies used for western blot and immunostaining

Antibody	Source and Catalog no.	Dilution	Analysis
MAFbx	ECM Biosciences # AP2041	1:1000	WB
MuRF1	R&D Systems #AF5366	1:1000	WB
Beclin1	Cell Signaling # 3495S	1:1000	WB
LC3b	Cell Signaling #2775S	1:1000	WB
Ubiquitin	Santa Cruz # sc-8017	1:1000	WB
IRE1alpha	Cell Signaling # 3294S	1:1000	WB
sXBP1 (E9V3E)	Cell Signaling # 40435	1:1000 / 1:50	WB / ChIP
PERK	Cell Signaling # 3192	1:1000	WB
CHOP	Cell Signaling # 2895S	1:1000	WB
Phospho-STAT3 (Y705)	Cell Signaling # 9145	1:1000	WB
STAT3	Cell Signaling # 30835	1:1000	WB
GAPDH	Cell Signaling # 2118	1:1000	WB
Anti-rabbit IgG HRP	Cell Signaling #7074S	1:2000	WB
Anti-mouse IgG HRP	Cell Signaling #7076S	1:2000	WB
Anti-goat IgG HRP	Invitrogen #A15999	1:2000	WB
Laminin	Sigma Chemical Co. # L9393	1:1000	IF
Myosin heavy chain (MyHC)	DSHB # MF20	1:100	IF
Perilipin-2	Proteintech # 15294-1-AP	1:300	IF
Anti-Mouse IgG2b AF555	Invitrogen # A21147	1:1000	IF
Anti-rabbit IgG AF555	Invitrogen #A31572	1:1000	IF

Table S2. List of primer sequences used for qPCR and ChIP assays.

Gene Name	Forward primer (5'-3')	Reverse primer (5'-3')
Xbp1 (exon2)	CCTGAGCCCGGAGGAGAA	CTCGAGCAGTCTGCGCTG
β-actin	CAGGCATTGCTGACAGGATG	TGCTGATCCACATCTGCTGG
Hspa5 (ChIP)	TGGTGGCATGGACCAATCAG	CGCCGACTCGCCTTATATAC
Dnajb9 (ChIP)	GAGCCGACCTACACGAAAC	AGGACCAAACGGCAACAA
Ern1	CCTTTGCTGATAGTCTCTGCCCAT	TTACCACCAGTCCATCGCCATT
Xbp1	TGTCCATTCCCAAGCGTGTTCT	TGGAGCAGCAAGTGGATTT
sXbp1	AAGAACACGCTTGGGAATGG	CTGCACCTGCTGCGGAC
Becn1	TGAAATCAATGCTGCCTGGG	CCAGAACAGTATAACGGCAACTCC
Map1lc3b	CTGGTGAATGGGCACAGCATG	CGTCCGCTGGTAACATCCCTT
Atg12	ACAAAGAAATGGGCTGTGGAGC	GCAGTAATGCAGGACCAGTTTACC
Fbxo32	GTCGCAGCCAAGAAGAGAAAGA	TGCTATCAGCTCCAACAGCCTT
Trim63	TACTGCATCTCCATGCTGGTG	TGGCGTAGAGGGTGTCAAACTT
Fbxo30	TCGTGGAATGGTAATCTTGC	CCTCCCGTTTCTCTATCACG
Ddit3	TGAAAGCAGAACCTGGTCCA	CACTGTTCATGCTTGGTGCA
Dnajb9	TTAGCCATGAAGTACCACCCTGAC	TTCCGACTATTGGCATCCGA
Edem1	CGGCTATGACAACTACATGG	GTTCAGATTGGACTCTC
Eif2ak3	ACTCCTGTCTTGGTTGGGTCTGAT	CGTGCTCCGCTTATTCCTTTCT
Bloc1s1	GCCTACATGAACCAGAGAAAG	GTTCTCCACCATTCCAATCC
Pdgfr	CCAAGTCAGGTCCCATTTAC	GGTCTTTCTTCGGCTTCTC
Scara3	GGGTTTCTATGGCTGGTTAG	TGCAGAGACCAGAGTAGTT
Sparc	CAACTGCAATTGGGCTTTC	ACCAGTCTCACTTCCTCTAC
Ppard	TCCATCGTCAACAAAGACGGG	ACTTGGGCTCAATGATGTCAC
Ppargc1a	TGGAGTGACATAGAGTGTGCTGC	CTCAAATATGTTCGCAGGCTCA
Cd36	GGCCAAGCTATTGCGACAT	CAGATCCGAACACAGCGTAGA
Acox1	GGATGGTAGTCCGGAGAACA	AGTCTGGATCGTTCAGAATCAAG
Acox2	CCTTCCTAGACCTGCTTCCC	TGTCCGTCATAACAGCCAAG
Acox3	CTTCTGAGAAACGGGGACAA	GCTCGGTAGGCACTAAGAGG
Sirt1	GACGATGACAGAACGTCACAC	CGAGGATCGGTGCCAATCA
Hif1a	TGAGCTTGCTCATCAGTTGC	CCATCTGTGCCTTCATCTCA
Hadhb	GCACTTTCGGGTTTGTTG	GTGTGAGCTGGAGTCTTATC
Irisin (Fndc5)	CACAGAATATATCGTCCATG	GTCACCTCATCTTTGTTCTT
Atg5	ATCAGACCACGACGGAGCGG	GGCGACTGCGGAAGGACAGA
Il6	CCTTCTTGGGACTGATGCTGG	GCCTCCGACTTGTGAAGTGGT
Pdk4	AAAGGACAGGATGGAAGGAATCA	TTTTCCTCTGGGTTTGCACAT
MAFbx (ChIP)	CCTCGGAAAACAAGGCGAG	GTCTCTTTGTTGCCGGAAGA
LC3b (ChIP)	GTTAACAGATGCTCGCCCAG	TGTGTGTCTCAGTCCGCAG

Atg5 (ChIP)	TTCCGAGTTCAGGCGCTC	GAACCAGAGTGAACCGCAG
Xbp1 (ChIP)	CCCGGGACTACAGGACCA	CCACCACCACCATAGCCA
Il6 (ChIP)	CTCATGCTTCTTAGGGCTAGC	GAGTGGGTGGGGCTGATT
Pdk4 (ChIP)	AAACAAGGACAAGTCTGGGC	TCACTAGAAAGGCCTGGCAC

Table S3. Average TPM values for PBS-injected *Xbp1*$^{fl/fl}$ mice

Gene	Avg. TPM	Gene	Avg. TPM	Gene	Avg. TPM	Gene	Avg. TPM	Gene	Avg. TPM
Ubqln4	40.560	Cdk16	60.291	Snx9	13.452	Ctsl	53.734	Acox1	30.195
Sec23a	21.400	Siah1a	5.633	Wwp2	4.556	Map1lc3b	79.470	Plin5	8.484
Ube2j1	13.683	Ripk2	1.086	Ppp2ca	21.888	Bnip3	150.388	Cyp4f13	6.202
Uggt1	4.801	Sox17	6.869	Abhd5	14.324	Edem2	3.000	Por	10.225
Hsp90ab1	511.050	Mylip	6.343	Adam9	18.235	Dnajb9	18.080	Abcd1	8.015
Eif2ak1	13.608	Zer1	9.286	Pink1	316.814	Rnf121	10.078	Acadsb	46.350
Sel1l	9.256	Prickle1	1.311	Igf1r	1.503	Get4	23.249	Ppard	9.168
Sar1a	63.835	Abca2	3.934	Atg4d	33.443	Dnajc10	7.647	Echdc1	8.069
Ube4b	14.744	Pan2	2.947	Atg101	27.022	Rnf5	17.772	Acox3	6.484
Nploc4	19.388	Mgrn1	21.404	Atg4b	9.843	Syvn1	7.863	Adipor2	27.850
Dnajb12	25.350	Cnot2	11.504	Pik3c3	7.219	Sgta	53.460	Acacb	42.548
Xbp1	28.959	Khsrp	6.982	Hif1a	4.634	Usp19	49.324	Crat	132.295
Dnaja2	86.389	Fto	31.122	Ctsb	92.448	Man1b1	5.761	Pex7	14.007
Sec31b	5.033	Pip4k2b	14.309	Ambra1	5.278	Ube2g2	46.501	Hacl1	6.454
Rad23a	80.304	Zc3h18	9.096	Smcr8	2.811	Stat5a	3.486	Acad10	1.680
Stt3b	26.017	Tpcn1	15.327	Usp8	5.099	Stat1	5.025	Gcdh	16.737
Eif2ak4	1.249	Huwe1	14.918	Atg9a	45.830	Mcl1	62.713	Ivd	65.615
Map2k7	27.178	Mid1	3.076	Tax1bp1	30.003	Csf2rb2	0.532	Slc25a17	6.535
Ubxn4	21.071	Cblb	2.069	Akt2	91.849	Csf2rb	0.465	Klhl25	1.580
Rad23b	40.924	Ubc	1111.328	Optn	29.451	Jak3	1.893	Dgat1	9.182
Ubqln1	19.482	Trim63	127.276	Mapk1	33.787	Il4ra	2.008	Pex5	10.411
Rab12	152.817	Fbxo32	27.393	Mtmr4	1.942	Tyk2	2.101	Tysnd1	6.776
Ddb1	59.740	Zswim8	12.448	Uvrag	2.447	Stam	4.011	Etfb	254.387
Zfp36l2	5.323	Supt5	22.064	Ddit4	27.123	Stat2	2.084	Scp2	125.223
Ube2o	6.575	Ube2b	372.150	Dapk3	14.855	Mtor	5.761	Adh5	46.870
Vps11	8.044	Cdc23	6.832	Bcl2l1	8.863	Osmr	1.292	Eci2	61.269
Fbxw8	6.031	Exosc8	7.630	Tbc1d15	10.628	Pik3r1	3.342	Echdc2	8.136
Sptlc2	3.754	Sting1	2.009	Foxo3	3.185	Il6ra	0.754	Akt1	23.419
Nub1	15.236	Ralb	4.303	Rab7	135.455	Stat6	7.432	Eci1	103.961
Clu	37.495	Slc25a5	36.903	Pik3r1	3.342	Aox1	4.489	Mlycd	48.059
Pip4k2c	4.647	Zc3hav1	1.664	Rhot2	32.676	Il6st	23.087	Decr1	38.108
Anapc5	259.659	Ubox5	1.903	Ulk1	18.231	Stat3	11.730	Auh	38.259
Ube3b	48.180	Nsf	4.456	Atg13	12.485	C1qtnf9	14.000	Acad12	6.596
Gga1	20.818	Il1b	0.209	Atg2a	4.942	Pdk4	138.439		
Upf1	9.204	Fxr1	203.387	Sqstm1	305.650	Ppargc1a	4.292		
Wdr24	4.826	Mlh1	4.466	Bnip3l	55.155	Cpt1b	115.817		
Trim68	3.692	Tardbp	66.167	Lamp1	201.108	Acadm	189.071		
